# *Mycobacterium iranicum* Infection in HIV-infected Patient, Iran

**DOI:** 10.3201/eid1910.130658

**Published:** 2013-10

**Authors:** Abdolrazagh Hashemi-Shahraki, Parvin Heidarieh, Samira Azarpira, Hasan Shojaei, Mohammad Hashemzadeh, Enrico Tortoli

**Affiliations:** Pasteur Institute of Iran, Tehran, Iran (A. Hashemi-Shahraki, S. Azarpira, M. Hashemzadeh); Alborz University of Medical Sciences, Karaj, Iran (P. Heidarieh);; Isfahan University of Medical Sciences, Isfahan, Iran (H. Shojaei);; San Raffaele Scientific Institute, Milan, Italy (E. Tortoli)

**Keywords:** Mycobacterium iranicum, nontuberculous mycobacteria, HIV-positive, HIV infection, Iran, bacteria, Tuberculosis and other mycobacteria

**To the Editor:** The species *Mycobacterium iranicum* was described in 2013 (*1*) on the basis of 8 clinical strains isolated in various countries (Iran, Italy, Greece, the Netherlands, Sweden, and the United States). Recently, the isolation of *M. iranicum* from the sputum of a woman also was reported (*2*). We report the isolation of this newly recognized species from an HIV-positive patient.

A scotochromogenic, rapidly growing strain was isolated in 2012 from respiratory specimens of an HIV-positive 44-year-old Iranian man with chronic pulmonary disease. The patient had been found to be HIV seropositive (viral load >1,000 copies/mL, CD4 lymphocyte count 120/µL) in 2004 when he was hospitalized because of fever, weight loss, and oral candidiasis. Treatment with antiretroviral drugs, including stavudine, lamivudine, and nevirapine, was begun. The patient rapidly improved; the fever disappeared, he gained weight, and he was discharged from the hospital. At a 6-month follow-up visit, viral load was 1,000 copies/mL and CD4 lymphocyte count was 420/µL. He continued to receive antiretroviral treatment until 2010 when treatment was discontinued because of its high cost.

The man was hospitalized again in 2012 with mild fever, weight loss, chronic chest pain, and nonproductive cough. At that time, the viral load was >100,000 copies/mL, and CD4 count was 5 lymphocytes/µL. Tuberculin skin test results were negative, radiograph of the chest showed no abnormalities, and routine cultures of sputum and blood were negative for common bacteria. Lactate dehydrogenase level (98 U/L [reference <600 U/L]) was within normal limits, whereas liver function was abnormal (alanine aminotransferase level 95 U/L [reference <36 U/L], L-aspartate aminotransferase level 85 U/L [reference <29 U/L], alkaline phosphatase 180 U/L [reference 44–147 U/L], and total bilirubin 1.4 mg/dL [reference 0.3–1mg/dl]). Antiretroviral therapy was resumed, which led to an increase in CD4 cells (205 lymphocytes/µL after 1 month). The examination by microscopy (Ziehl-Neelsen staining) of 3 sputum samples did not reveal acid-fast bacilli; culture for mycobacteria was not done. Oral treatment with tetracycline was started, but the patient’s fever and chest pain remained unchanged.

After bronchoscopy, 2 of 3 bronchial lavage (BAL) samples were found to be positive for acid-fast coccobacilli by microscopy, and rapidly growing, deep orange mycobacteria grew in all 3 cultures. Giemsa stain did not show *Pneumocystis jirovecii* in BAL samples. A standard antituberculosis regimen was undertaken but did not result in substantial improvement. At 1 month follow-up, 1 sputum sample was negative for acid-fast organisms, and 1 BAL specimen was positive by microscopy and in culture. When the isolate was identified as *M. iranicum*, therapy was replaced with a combination of amikacin and ciprofloxacin for 3 months (standard treatment used in Iran for infections caused by rapidly growing mycobacteria), and the patient improved rapidly. Mycobacteria were neither observed nor grew in culture in a BAL specimen obtained 1 month after the change in therapeutic regimen.

Identification of the isolates was initially attempted with biochemical tests, and they were negative for niacin production, nitrate reduction, Tween 80 hydrolysis, and semiquantitative catalase. The tests were positive for urease activity, iron uptake, tellurite reduction, arylsulfatase (3 days after the start of the test), 5% NaCl tolerance, and heat-stable (68°C) catalase. The genetic sequencing of almost-complete (1,450 bp) 16S rRNA gene (*3*), a 710-bp fragment of the β-subunit of the RNA polymerase gene (*4*), and the hypervariable region (402 bp) of the 65-kDa heath-shock protein (*5*) revealed 99.8%, 99.4%, and 100% identity, respectively, with sequences of the type strain found in GenBank and definitively confirmed the identification.

The clinical criteria required by the American Thoracic Society and Infectious Disease Society of America (*3*) to assess the importance of the isolation of a nontuberculous mycobacterium from pulmonary specimens include, in adjunct to a specific symptomatology, the presence of nodular or cavitary lung lesions and the exclusion of any other possible cause of the disease. The normal thoracic radiograph findings for the case-patient described here cannot, however, be considered a definitive exclusion criterion: in highly immunocompromised patients, a chest radiograph may show no abnormalities, even when substantial pathologic features of infection are present (*6*). The microbiological criteria were clearly fulfilled by isolating the organism from multiple sputum specimens and the BAL specimens. The patient’s response to the treatment and the disappearance of thoracic symptoms further support the assertion.

Our report confirms the potential pathogenicity of *M. iranicum*. In addition to the case described here, 9 isolations of this species have been reported so far. Among them, the clinical relevance has been demonstrated for 2 strains grown from respiratory specimens of patients with pulmonary disease and for 1 strain isolated from a cutaneous lesion (*1**,**2*). The role of an accurate identification, in conjunction with symptoms and radiographic findings, is central to understanding the clinical significance of mycobacteria isolated from pathologic specimens.
